# Missed opportunities in post-total knee arthroplasty rehabilitation in Indonesia: a narrative review of insurance-driven delays in functional recovery

**DOI:** 10.3389/fresc.2026.1767231

**Published:** 2026-04-10

**Authors:** Yose Waluyo, Nuralam Sam, Yukio Mikami, Fikri Rifa Hamdi

**Affiliations:** 1 Department of Physical Medicine and Rehabilitation, Faculty of Medicine, Hasanuddin University, Makassar, Indonesia; 2 Department of Rehabilitation, Hiroshima University Hospital, Hiroshima, Japan

**Keywords:** delayed functional recovery, early mobilization, health system barriers, Indonesia, insurance coverage, knee arthroplasty, postoperative rehabilitation

## Abstract

Total knee arthroplasty (TKA) is increasingly performed worldwide, including in Southeast Asia and Indonesia, and early-phase rehabilitation is a primary determinant of postoperative functional recovery; delays during this critical window can impair strength, mobility, independence, and long-term quality of life. This narrative review examines the gap between internationally established evidence-based rehabilitation protocols and current practices in Indonesia and describes the impact of insurance-related delays on functional outcomes. Global literature consistently supports early mobilization, intensive therapeutic exercise, multidisciplinary care, and standardized outcome monitoring, whereas rehabilitation in Indonesia is often constrained by short hospital stays, uneven distribution of rehabilitation professionals, the absence of national protocols, and, most critically, limited BPJS insurance coverage. These constraints create missed opportunities during the first 1–2 postoperative weeks, contributing to stiffness, muscle weakness, abnormal gait, delayed daily function, and lower patient satisfaction, while systemically increasing long-term care needs, caregiver burden, and indirect healthcare costs. Because functional recovery after TKA is highly dependent on timely, intensive early rehabilitation, administrative and insurance-related barriers in Indonesia produce clinically meaningful delays and long-term dependency. By mapping these systemic gaps and their consequences, this review aims both to inform future policy refinement and to raise awareness among policymakers, clinicians, and stakeholders regarding the urgent need to optimize early rehabilitation to improve outcomes and strengthen healthcare system efficiency.

## 1 Introduction

End-stage knee osteoarthritis (KOA) is a major cause of disability worldwide, and treatment typically follows a stepped care model in which total knee arthroplasty (TKA) becomes the final option when conservative measures fail. TKA is well established as an effective intervention for restoring pain-free mobility, and its utilization continues to rise globally as populations age. For example, more than 21,000 TKAs were performed in the Netherlands in 2021, with higher rates among older adults and women (1). Similar trends are observed across Southeast Asia and Indonesia, where TKA is most commonly performed in older adults, women, and individuals with multiple comorbidities (2). Cultural expectations, particularly the demand for deep knee flexion in many Asian societies, further shape postoperative functional goals (3).

In the Asia–Pacific region, postoperative rehabilitation practices vary widely. Hospital stays are generally longer than in Western countries, typically ranging from 5 to 10 days, yet the extended stay does not consistently translate into structured, evidence-based rehabilitation programs (4). At the same time, cultural expectations for greater knee flexion often require more intensive and targeted rehabilitation approaches (3).

Beyond surgical technique and implant quality, the success of TKA depends heavily on postoperative rehabilitation. Systematic reviews consistently demonstrate that physiotherapy and therapeutic exercise accelerate functional recovery, improve quadriceps strength, and reduce pain (5, 6). Community- and home-based rehabilitation programs have also shown comparable outcomes to hospital-based care when guided by trained professionals (7). These findings highlight the essential role of early and intensive rehabilitation in determining postoperative achievement of functional milestones.

However, the implementation of such rehabilitation protocols in Indonesia remains suboptimal. Although national medical service standards acknowledge the need for post-TKA rehabilitation, no unified national protocol exists (8). Limited access to rehabilitation services due to the uneven distribution of physiatrists and physiotherapists further restricts care availability (9). Moreover, the national insurance system (BPJS) reimburses only a limited number of physiotherapy sessions, causing many patients to miss the critical early rehabilitation window (10).

These conditions create a phenomenon best described as an insurance-driven delay, in which functional recovery is hindered not by medical complications but by financial and administrative constraints. Evidence from case studies and prior reviews shows that delayed early rehabilitation can lead to significant quadriceps weakness, restricted range of motion, and poorer long-term outcomes (11). This narrative review therefore aims to map these missed opportunities within Indonesia's post-TKA rehabilitation landscape by examining gaps between evidence-based protocols and real-world practice, identifying systemic barriers, and outlining the clinical consequences of insurance-related delays.

## 2 Methods

This narrative review synthesizes global evidence on postoperative rehabilitation following total knee arthroplasty (TKA) and contextualizes these findings within the Indonesian healthcare system. A structured literature search was conducted in PubMed, Scopus, Google Scholar, and relevant governmental or organizational policy repositories using combinations of the following keywords: “total knee arthroplasty,” “postoperative rehabilitation,” “early mobilization,” “insurance coverage,” “health financing,” “delayed recovery,” “BPJS Indonesia,” and “Asia-Pacific rehabilitation systems.”

The search covered publications from 2010 to 2025, prioritizing recent clinical guidelines, randomized trials, meta-analyses, rehabilitation protocols, and policy documents related to postoperative TKA care. Studies were included if they met one or more of the following criteria:

1. described evidence-based rehabilitation protocols after TKA;
2. examined clinical outcomes associated with early vs. delayed rehabilitation;
3. evaluated systemic, administrative, or insurance-related barriers to postoperative care; or
4. provided national or regional data relevant to Indonesia or comparable low- and middle-income contexts.

Exclusion criteria included studies unrelated to postoperative rehabilitation, publications focused solely on surgical technique or implant design, and commentary pieces lacking empirical support. Additional gray literature-such as BPJS insurance regulations, professional society recommendations, and Ministry of Health workforce data-was reviewed to provide a broader understanding of Indonesia's rehabilitation infrastructure.

Findings were synthesized narratively, with emphasis on thematic integration across clinical, systemic, and policy domains, rather than quantitative pooling, due to heterogeneity in study designs and outcomes.

## 3 Evidence-based rehabilitation protocols after TKA

### 3.1 Goals of rehabilitation

Post-TKA rehabilitation aims to reduce pain, restore knee range of motion, strengthen lower-limb musculature, normalize gait, and facilitate independence in daily activities, all of which are core determinants of long-term surgical success (6). Systematic reviews consistently show that structured rehabilitation accelerates pain reduction, improves range of motion, and enhances quadriceps strength, a primary contributor to knee function (5, 6). Early high-intensity exercise produces superior outcomes compared with lower-intensity protocols without increasing adverse events (12).

International guidelines, including the American Physical Therapy Association (APTA) Clinical Practice Guideline, emphasize early mobilization, progressive functional exercise, and adjunctive therapies such as neuromuscular electrical stimulation (NMES) to improve early activation of the quadriceps (13). Community-based and home-based rehabilitation programs can also achieve comparable outcomes when delivered systematically with appropriate professional supervision, supporting continuity of care after hospital discharge (7, 14).

Globally, there is strong consensus that early, intensive rehabilitation is essential for achieving optimal physical, functional, and quality-of-life outcomes after TKA. In Indonesia, however, these goals are often unmet due to limited insurance-covered physiotherapy sessions and the absence of a national post-TKA rehabilitation protocol, resulting in missed early recovery windows and suboptimal long-term functional outcomes (10).

### 3.2 Typical protocols in advanced health systems

High-income countries, including the United States, Japan, Singapore, and Australia, have established relatively uniform rehabilitation pathways after TKA. Core principles include early mobilization, high-frequency therapy, multidisciplinary involvement, and standardized functional outcome monitoring (15).

Mobilization typically begins within the first 24 h after surgery and, in some centers, on the same day as the procedure (16). Early exercises include quadriceps sets, ankle pumps, and active knee flexion–extension, followed by standing and ambulation training with assistive devices (17).

In the Asia-Pacific region, many centers have adopted accelerated rehabilitation approaches with mobilization initiated on the day of surgery or the next day. Some facilities report intensive interval-based rehabilitation programs lasting up to eight hours per day during the acute phase. Modalities such as continuous passive motion (CPM), manual therapy, dynamic extension splints, and NMES are commonly used. However, unlike Western systems that emphasize early discharge and home-based rehabilitation, many Asia-Pacific countries continue to rely on hospital-based programs, often paid out-of-pocket and therefore less accessible (4).

High-income centers also deliver greater training frequency and intensity than typical Indonesian practice. For example, Brigham and Women's Hospital provides twice-daily inpatient physiotherapy, followed by intensive outpatient sessions for several weeks (17). Australian rapid-recovery programs combine same-day mobilization, multimodal analgesia, and early transition to self-directed cycling-based exercises (18).

Most established protocols set clear early targets: achieving ≥90° of knee flexion within 2–3 weeks, independent ambulation within 5–7 days, and progression to advanced functional training by weeks 6–12 (15, 19). Standardized outcome measures such as WOMAC, KSS, OKS, KOOS, LEFS, and functional tests like the Timed Up and Go (TUG) and 6-Minute Walk Test (6MWT) are routinely used to monitor progress (20).

Despite variations in specific techniques, global post-TKA rehabilitation consistently prioritizes very early mobilization, daily intensive training, early ROM milestones, and structured monitoring, all of which stand in sharp contrast to the limited therapy frequency, inconsistent protocols, and insurance-driven restrictions commonly encountered in Indonesia.

### 3.3 Expected outcomes when protocols are followed

When evidence-based rehabilitation protocols are implemented consistently, patients achieve predictable and favorable outcomes. Knee flexion of ≥90° is typically reached within 2–3 weeks, enabling essential functional activities such as sitting, standing, and walking (19). Most patients regain independent ambulation within 5–7 days, especially when early mobilization is initiated on postoperative day one (13).

Early high-intensity rehabilitation accelerates quadriceps recovery, which is essential for knee stability and functional performance, and improves objective outcomes such as TUG, 6MWT, and WOMAC and KSS scores (6, 12). Structured rehabilitation programs also improve quality of life, with patients reporting faster pain reduction, greater independence, and higher satisfaction levels (18). Community-based models achieve comparable improvements when programs are standardized and outcomes are monitored (7).

Collectively, these findings affirm that optimal post-TKA outcomes depend on early, intensive rehabilitation targeting ROM, strength, ambulation, and functional mobility. In Indonesia, however, these milestones are frequently missed due to short inpatient stays, limited therapy intensity, and restricted BPJS insurance coverage, leading to delayed recovery and diminished long-term outcomes (21).

## 4 Systemic barriers to optimal rehabilitation in Indonesia

### 4.1 Hospital stay duration

The duration of hospitalization after TKA plays an important role in facilitating early rehabilitation. Longer inpatient stays allow earlier mobilization, restoration of knee motion, and preparation for functional independence before discharge (22). In Indonesia, evidence on post-TKA length of stay remains limited. A study from Surabaya reported an average of 5.58 days with discharge criteria based on assisted ambulation and stable surgical wounds (23). A study from Bali described patient characteristics without reporting hospitalization duration (2), underscoring the need for more comprehensive national data.

Across the Asia–Pacific region, postoperative stays typically range from 5 to 10 days, which is longer than the 2 to 3 days commonly observed in the United States. However, extended hospitalization in many Asian settings does not consistently include structured, evidence-based rehabilitation, limiting its impact on accelerating recovery (4). In Australia, rapid-recovery programs have reduced inpatient stays from 7.1 days in 2009 to 5.4 days in 2016 through same-day mobilization and early transition to home-based rehabilitation. The United States has achieved even shorter stays, declining from 4.06 days in 2002 to 2.97 days in 2013 (18).

Recent evidence from Japan provides an important contrast illustrating how insurance structures shape postoperative LOS and the volume of rehabilitation delivered. Under Japan's fee-for-service system, hospitals are financially incentivized to maintain extended inpatient care, resulting in a median LOS of 31 days with at least two hours of structured rehabilitation daily. In contrast, Indonesia's fixed BPJS reimbursement discourages prolonged hospitalization and covers only limited outpatient physiotherapy, resulting in far fewer rehabilitation opportunities following a 2–5-day LOS. This comparison suggests that Indonesia's challenge lies less in the short LOS itself and more in a financing system that restricts rehabilitation intensity during the critical early recovery phase (24).

Thus, although hospitalization durations in Indonesia are broadly comparable to global norms, the absence of intensive, structured early rehabilitation during this period leads to missed opportunities for functional recovery. Many patients are discharged before achieving essential milestones, with no immediate transition to high-frequency outpatient or community-based therapy to compensate for this early gap.

### 4.2 BPJS insurance policy constraints

Indonesia's national health insurance system (BPJS Kesehatan) is designed to promote equitable access, yet its reimbursement structure imposes substantial limitations on post-TKA rehabilitation. The most significant barrier is the narrow coverage of outpatient physiotherapy, which typically allows only two sessions per week, far below the 3 to 5 supervised sessions per week commonly provided in the United States and Australia during the first month of recovery (13). BPJS also does not finance structured subacute rehabilitation or community-based programs, which are standard in countries such as Singapore, leaving many patients, particularly those outside major urban centers, without continuity of care during the early period when rehabilitation has the highest potential impact (7).

Administrative requirements further delay initiation of therapy. After discharge, patients must return to the orthopedic clinic before being re-referred to rehabilitation, and regulations prevent same-day treatment following the initial assessment. These steps commonly introduce several days to weeks of delay before outpatient therapy can begin.

In contrast, Japan's fee-for-service system financially incentivizes hospitals to extend inpatient care, resulting in a median postoperative LOS of 31 days with at least two hours of structured rehabilitation provided daily (24). Indonesia's fixed INA-CBG reimbursement discourages prolonged hospitalization and restricts outpatient coverage, producing a markedly lower cumulative therapy dose across the early postoperative phase.

Together, these financial and administrative constraints create clear insurance-driven delays, limiting rehabilitation intensity precisely when it is most critical for achieving optimal functional recovery.

### 4.3 Healthcare workforce distribution

The availability and distribution of rehabilitation professionals are critical for effective post-TKA care. International protocols emphasize multidisciplinary teams led by physical medicine and rehabilitation specialists, supported by physiotherapists, orthopedic surgeons, and trained nursing staff (13). In Indonesia, however, limited workforce capacity and marked geographic imbalances significantly restrict service delivery.

According to national workforce data, Indonesia's 1,484 physiatrists serve approximately 282 million people and 3,217 hospitals, an insufficient ratio for a country of this size and geographic complexity. On average, this translates to one physiatrist for every 190,000 residents and one physiatrist for every two hospitals, with considerably more severe shortages outside Java. In regions such as Kalimantan, Maluku, Nusa Tenggara, and Papua, where hospital availability is already low and individual facilities may serve 95,000 to 116,000 people, the scarcity of rehabilitation specialists further limits access to comprehensive postoperative care. These workforce gaps demonstrate that Indonesia remains well below the capacity needed to deliver timely, intensive, and equitable rehabilitation services, particularly for high-demand postoperative conditions such as TKA (9).

This pattern reflects broader trends in low and middle-income countries, where urban–rural disparities consistently undermine rehabilitation access (16). Indonesia faces the same structural challenge: specialist services are concentrated in urban centers, while rural and remote areas lack the personnel required to provide early, intensive rehabilitation during the critical postoperative window.

### 4.4 Absence of a national post-TKA rehabilitation protocol

Although the 2019 National Medical Service Standards acknowledge the importance of musculoskeletal rehabilitation, they do not include a dedicated section on post-TKA management (8). Existing guidelines reference conditions such as knee osteoarthritis but do not provide structured rehabilitation pathways for arthroplasty patients.

As a result, rehabilitation practices vary significantly across hospitals. Larger tertiary centers with adequate facilities and personnel may deliver relatively intensive programs, whereas smaller hospitals often provide only minimal intervention. These disparities are compounded by BPJS coverage limitations, which prevent uniform implementation even when clinical need is evident.

Unlike high-income countries that have standardized national guidelines, such as the APTA Clinical Practice Guideline in the United States or rapid recovery pathways in Australia, Indonesia lacks a formal consensus on post-TKA rehabilitation. This absence contributes to inconsistent service delivery and frequent loss of early rehabilitation opportunities due purely to institutional policy differences.

Overall, the lack of a standardized national protocol not only results in variability of care but also delays functional recovery at a systemic level, particularly when combined with insurance and workforce constraints.

## 5 Insurance-driven delayed functional recovery: clinical consequences

### 5.1 Definition and mechanisms of delayed recovery

Delayed recovery after TKA refers to the failure to achieve expected functional milestones due to interruptions in early rehabilitation. These delays are not caused by medical complications but by systemic barriers, primarily insurance limitations and administrative regulations, that prevent patients from receiving timely and intensive therapy.

This phenomenon is best described as an insurance-driven delay, in which functional progress is hindered by BPJS reimbursement caps, lengthy administrative pathways, and the absence of structured subacute rehabilitation within the national insurance scheme (10, 21).

Several mechanisms contribute to this delay. First, patients experience a therapy gap during the critical first 1–14 days after surgery, when daily or near-daily rehabilitation is required (11, 25). In Indonesia, outpatient physiotherapy often begins only after multiple administrative steps, postponing therapy by days or weeks.

Second, delayed rehabilitation impairs early quadriceps activation and slows recovery of knee motion. Evidence shows that neuromuscular electrical stimulation (NMES) within the first 48 h can preserve quadriceps strength, but postponed initiation increases the risk of prolonged weakness (26). Third, early rehabilitation is essential for neuromuscular and behavioral adaptation. Loss of early training opportunities leads to persistent gait abnormalities, compensatory movement patterns, and reduced adherence to exercise programs (4).

Thus, delayed recovery in Indonesia arises primarily from non-medical, insurance-driven constraints that limit access to early, intensive therapy when it is most critical.

### 5.2 Clinical manifestations

Insurance-driven delays manifest in several key functional deficits. Early stiffness and restricted knee range of motion (ROM) are common. Achieving approximately 80° of flexion within 1–2 weeks predicts the ability to reach ≥110° by weeks 7–8, but delayed rehabilitation hinders this progression and increases the likelihood of persistent stiffness (1).

Quadriceps weakness, which plays a central role in knee stability and gait, is further exacerbated when early activation and NMES are not provided. Persistent weakness slows functional recovery and limits mobility (6).

Gait abnormalities, including antalgic gait and dependency on assistive devices, may become entrenched when gait training is not initiated promptly. These maladaptive motor patterns are difficult to correct once established (11). Functional independence is also affected. Delayed rehabilitation slows progress in basic activities such as stair climbing, sit-to-stand transitions, and household tasks. These limitations reduce overall quality of life, with lower patient-reported outcomes observed when early intensive therapy is not provided (27).

Collectively, these clinical manifestations underscore the impact of missed early rehabilitation on stiffness, ROM limitation, quadriceps weakness, abnormal gait, and delayed independence.

### 5.3 Missed window of opportunity

The first two postoperative weeks represent a narrow window during which muscles, nerves, and motor behavior are most responsive to rehabilitation stimuli. Delayed initiation during this phase leads to lost neuromuscular momentum, slower functional gains, and greater risk of long-term maladaptation (27).

A similar pattern is observed in the Asia–Pacific region, where longer hospital stays do not necessarily translate into structured rehabilitation programs, resulting in the same missed opportunities despite greater inpatient duration (4). The early postoperative period provides biological and behavioral advantages that cannot be fully recaptured once lost.

### 5.4 Economic and systemic impacts

Insurance-driven delays not only slow functional recovery but also increase long-term healthcare utilization. Patients with stiffness, weakness, or gait dysfunction often require additional physiotherapy sessions, prolonged use of assistive devices, or even revision procedures, all of which contribute to higher direct and indirect costs (25).

Limited therapy intensity also reduces patient satisfaction, as individuals consistently rate structured, intensive rehabilitation more effective than restricted programs (6, 14). Satisfaction is an important indicator of system quality and influences adherence and outcomes.

At the national level, BPJS restrictions intended to improve efficiency may paradoxically increase long-term expenditures. Inadequate early rehabilitation prolongs functional dependence, diminishes return-to-work potential, and amplifies the economic burden on families (10). In contrast, countries with structured early rehabilitation, such as Australia, demonstrate faster recovery and reduced long-term healthcare costs (18).

### 5.5 Implication for practice and health systems

This review underscores that delays in post–TKA rehabilitation represent system-level challenges rather than isolated clinical shortcomings. Early inpatient mobilization alone is insufficient when not followed by timely and adequately intensive rehabilitation after discharge. Interruptions during the early post-discharge period may diminish initial functional gains, highlighting the critical role of continuity across acute and post-acute phases of care.

From a health system perspective, insurance-driven delays reveal a misalignment between rehabilitation delivery and biological recovery windows. Improving continuity between inpatient care and subsequent rehabilitation, through better coordination or alternative delivery models, may help preserve early recovery gains and reduce long-term functional dependence. These findings reinforce the need to conceptualize rehabilitation as a continuous episode of care, particularly within resource-constrained health systems.

## 6 Discussion

Post-TKA rehabilitation is a critical determinant of surgical success, functioning as an essential component alongside operative technique and implant quality. International evidence consistently demonstrates that early mobilization, high-intensity exercise, multidisciplinary involvement, and standardized functional assessments yield superior outcomes (13, 17). High-income countries such as the United States, Australia, and Singapore have developed relatively uniform rehabilitation pathways that integrate inpatient, outpatient, and community-based care, enabling faster functional recovery, higher patient satisfaction, and long-term cost efficiency (14).

In contrast, rehabilitation practices in Indonesia remain highly variable and are often constrained by low therapy frequency, administrative delays, and restricted insurance coverage, which together highlight a substantial gap between global best practices and real-world implementation. Similar challenges are also observed in other low and middle-income countries, where limited financing structures and fragmented service delivery contribute to reduced therapy intensity during the early postoperative phase.

Growing evidence suggests that alignment between surgical timing and early initiation of physiotherapy plays a critical role in optimizing functional recovery following total knee arthroplasty. Early mobilization within the first postoperative hours has been associated with improved quadriceps activation, earlier gait restoration, and reduced postoperative complications (13). In systems where same-day or 24-hour discharge pathways are implemented, close coordination between surgical and rehabilitation teams is essential to ensure that early discharge does not compromise functional progression.

Surgical scheduling may also influence the success of early mobilization and same-day or 24-hour discharge pathways following total knee arthroplasty. Evidence suggests that procedures initiated later in the day may reduce the likelihood of successful same-day discharge due to logistical constraints and delayed achievement of early ambulation goals. For instance, Shen et al. reported that a procedure start time after 11:00 AM was significantly associated with failed same-day discharge following primary TKA (28). Similarly, within an enhanced recovery after surgery (ERAS) pathway, Lee et al. demonstrated that patients whose operations were completed in the morning and who received physiotherapy review on the same afternoon were most likely to achieve discharge within 24 h. These findings highlight the importance of synchronizing surgical scheduling with early postoperative physiotherapy to maximize the early rehabilitation window (29).

In many Indonesian hospitals, surgical scheduling is often influenced by operating room availability, administrative processes, and insurance authorization pathways. These systemic factors may affect the coordination between surgical procedures and early postoperative rehabilitation services.

In the Indonesian healthcare context, however, such synchronization remains limited. Administrative processes and insurance-related constraints may delay the initiation of physiotherapy beyond the critical early postoperative window. Consequently, patients may forfeit a crucial period for neuromuscular activation and functional retraining, thereby reinforcing the gap between surgical efficiency and rehabilitation intensity during the early recovery phase.

A central barrier in Indonesia is the BPJS financing structure. Although designed to ensure equitable access, its limited physiotherapy coverage, which typically allows only two sessions per week, and the absence of structured subacute rehabilitation result in unintended under-rehabilitation, particularly during the period when therapy intensity has the greatest influence on outcomes (10, 30). Comparatively, insurance design plays a decisive role across healthcare systems. Japan's fee-for-service model, for instance, incentivizes prolonged inpatient care, resulting in a median length of stay of 31 days with two hours of structured rehabilitation daily, which enables patients to reach key functional milestones more reliably before discharge (24). This stands in stark contrast to Indonesia's fixed INA-CBG reimbursement, which discourages extended hospitalization and limits outpatient therapy, ultimately producing a much lower cumulative rehabilitation dose during the critical early recovery window.

Beyond surgical timing and insurance-related limitations, the concept of Enhanced Recovery After Surgery (ERAS) offers a structured framework for optimizing perioperative care. ERAS pathways in total knee arthroplasty integrate multimodal analgesia, early mobilization, and coordinated rehabilitation to accelerate functional recovery and reduce postoperative complications. However, in the Indonesian healthcare context, ERAS adoption remains fragmented and inconsistently implemented. Insurance-driven authorization procedures, limited reimbursement for intensive early physiotherapy, and the absence of bundled perioperative payment models hinder the seamless integration of rehabilitation within surgical pathways. As a result, early physiotherapy, an essential determinant of surgical success, is frequently delayed or reduced in intensity, diminishing the functional gains achievable during the critical early recovery window. Integrating ERAS principles into national clinical pathways and reimbursement structures may therefore represent a strategic opportunity to align financial incentives with evidence-based rehabilitation practices.

The gap in Indonesia is further exacerbated by poor integration between discharge planning and community-based rehabilitation. In high-income systems, rehabilitation pathways are established before discharge and include seamless transitions into outpatient or subacute care. In Indonesia, however, patients are typically discharged within 2–5 days and must navigate multiple administrative steps before therapy can resume. Rural patients face even greater barriers due to limited service availability, leading to discontinuity of care and prolonged functional impairment. Thus, the core problem extends beyond therapy quotas to the absence of a coordinated rehabilitation continuum across service levels.

The resulting challenges are multidimensional. Clinically, delayed rehabilitation leads to stiffness, quadriceps weakness, gait abnormalities, and delayed independence in daily activities (11, 25). Systemically, insurance restrictions and uneven workforce distribution lead to increased long-term therapy needs, reduced patient satisfaction, and diminished productivity (1). This reflects a broader paradox seen in many emerging economies: financing policies designed to widen access may inadvertently compromise functional outcomes by allowing patients to miss the early rehabilitation window.

This narrative review does not propose direct policy reforms; rather, it delineates systemic barriers and their consequences. By mapping disparities between evidence-based rehabilitation protocols and current Indonesian practice, this review highlights the need for greater recognition of insurance-driven delays and their substantial impact on patient recovery. Future work should explore alternative rehabilitation models, strengthen discharge-to-community integration, and evaluate financing mechanisms to support adequate therapy intensity. Building awareness of these challenges is an essential first step toward collaborative clinical and policy efforts to strengthen Indonesia's post-TKA rehabilitation system.

## 7 Strengths and limitations

This narrative review provides a comprehensive synthesis of global evidence on post-TKA rehabilitation while contextualizing it within Indonesia's unique healthcare landscape, an approach that is rarely addressed in the existing literature. Its primary strength lies in highlighting insurance-driven delays as a distinct and under-recognized mechanism influencing functional recovery, integrating clinical, systemic, and policy dimensions into a single conceptual framework. However, this review is limited by the heterogeneity of available Indonesian data, the absence of national rehabilitation registries, and the reliance on observational and region-specific studies. As a narrative review, it does not include systematic data extraction or quantitative pooling, which may restrict the generalizability of the synthesized findings.

## 8 Future directions

Future research should evaluate the feasibility and effectiveness of early intensive rehabilitation models tailored to Indonesia's health system, including structured home-based programs, community rehabilitation pathways, and the integration of telerehabilitation to improve continuity of care. Prospective cohort studies or pragmatic trials assessing therapy frequency, early mobilization strategies, and the impact of reducing administrative delays are needed to strengthen the evidence base. Additionally, health economic evaluations examining the long-term cost implications of restricted physiotherapy coverage would provide valuable insights for policy stakeholders. Establishing national guidelines and rehabilitation registries may further support consistent implementation and monitoring of post-TKA care across diverse healthcare settings.

## 9 Conclusion

Optimal functional recovery after TKA relies heavily on early and intensive rehabilitation. Global evidence consistently shows that early mobilization, daily therapeutic exercise, and coordinated multidisciplinary care accelerate postoperative recovery, while inadequate or delayed intervention increases the risk of stiffness, quadriceps weakness, gait abnormalities, and loss of independence. When early rehabilitation is insufficient, patients often develop long-term functional limitations that require prolonged or recurrent rehabilitation, sometimes extending for months or even years, which ultimately reduces quality of life and increases dependence on caregivers.

In Indonesia, systemic constraints, particularly BPJS funding limits, restricted outpatient coverage, and multi-step administrative pathways, create insurance-driven delays that hinder recovery during the phase when rehabilitation has the greatest impact. Rather than proposing direct policy reform, this narrative review seeks to increase awareness among policymakers, institutional leaders, and multidisciplinary healthcare providers about these gaps and their clinical consequences. Building a shared understanding of these issues is a crucial first step toward improving therapy intensity, strengthening patient independence, and enhancing the long-term efficiency of Indonesia's healthcare system.
